# Letter to the Editor: Comments on “Association between the ICAM-1 gene polymorphism and coronary heart disease risk: a meta-analysis”

**DOI:** 10.1042/BSR20190554

**Published:** 2019-05-17

**Authors:** Morteza Gholami, Mahsa M. Amoli, Farshad Sharifi

**Affiliations:** 1 Metabolic Disorders Research Center, Endocrinology and Metabolism Molecular-Cellular Sciences Institute, Tehran University of Medical Sciences, Tehran, Iran; 2 Endocrinology and Metabolism Research Center, Endocrinology and Metabolism Clinical Sciences Institute, Tehran University of Medical Sciences, Tehran, Iran; 3 Elderly Health Research Center, Endocrinology and Metabolism Population Sciences Institute, Tehran University of Medical Sciences, Tehran, Iran

**Keywords:** Coronary artery disease, Comment, ICAM-1, Meta-Analysis, Polymorphism

## Abstract

Yin et al. (*Bioscience Reports* (2019) **39**, BSR20180923) recently published a meta-analysis about the association between the K469E (rs5498) polymorphism and risk of coronary heart disease (CHD). Authors included 14 studies based on their inclusion criteria. They indicated that only studies which their genotyping data were in Hardy–Weinberg equilibrium (HWE) were included in their meta-analysis. They also tested HWE for these studies and found all the control groups in HWE. As their main finding, they concluded that ‘K469E polymorphism is associated with CHD risk and the K allele is a more significant risk factor for developing CHD amongst Chinese and Caucasians populations’. However, there seems to be presenting some mistakes in HWE test which strongly affects included studies and the final conclusion. Here we aim to comment on the issue.

Dear Editor,

Unfortunately, based on our analysis, contrary to meta-analysis by Yin et al. [1], studies they included in their meta-analysis were not in Hardy–Weinberg equilibrium (HWE), and many included articles (seven articles) show deviation from HWE, even after adjustment. It seems that authors made some mistake in calculating HWE. In Table 1 we showed *P*-values for HWE test and ineligible studies, based on ‘HardyWeinberg’ package in R programming language (https://cran.rproject.org/web/packages/HardyWeinberg/HardyWeinberg.pdf). Our results were double checked with STATA (genhwi form of genhw, https://www.stata.com/users/mcleves/genhw/genhw.hlp), and also manually. In manual method, *P*-value of HWE test was calculated based on four following steps. (i) We calculated allele frequencies in control group: K = [(2 × KK) + KE]/(2 × total), so E should be E = 1 − K. (ii) We calculated expected genotypes based on allele frequencies: KK = K^2^ × total, KE = (2 × K × E) × total, and EE = EE^2^ × total. (iii) We carried out chi-square test between observed and expected genotypes (χ^2^ = Σ(Ob **−** Ex)^2^/Ex). (iv) Finally, results were interpreted based on chi-square routine distribution table (steps (i–iii) are shown in Table 2 and step (iv) in Table 3). Also regarding the study by Sarecka-Hujar et al. [2], the genotyping data were not correctly included in Table 1 of their meta-analysis, GG(EE) and AA(KK) genotypes and allele frequencies were displaced in both case and control groups. Correct data are shown in Table 1. Also, they [2] indicate that ‘the distribution of ICAM1 genotypes was not compatible with HWE’ which clearly violates inclusion criteria (iv) in Yin et al. [1] meta-analysis.

**Table 1:** Genotyping data and HWE results for studies in Yin et al. [1] meta-analysis

Studies	Case KK	KE	EE	Control KK	KE	EE	*P*-value	Adjusted *P*-value	Design
Shang, Q. (2005)	48	50	24	29	33	35	0.002	0.005	Exclude
**Li, Y.J. (2010)**	**47**	**39**	**7**	**52**	**36**	**13**	**0.103**	**0.180**	**Include**
Lu, F.H. (2006)	61	69	30	45	65	59	0.003	0.008	Exclude
**Zhang, S.R. (2006)**	**111**	**52**	**10**	**69**	**59**	**13**	**0.940**	**0.973**	**Include**
Rao, D. (2005)	84	41	20	59	19	66	<0.001	<0.001	Exclude
**Wei, Y.S. (2006**)	**124**	**84**	**17**	**101**	**103**	**26**	**0.973**	**0.973**	**Include**
Zhou, Y.L. (2006)	38	45	20	102	62	33	<0.001	<0.001	Exclude
**Wang, M. (2005**)	**96**	**61**	**8**	**91**	**90**	**18**	**0.524**	**0.734**	**Include**
Jiang, H. (2002)	202	226	100	60	66	87	<0.001	<0.001	Exclude
**Milutinović, A. (2006)**	**47**	**72**	**33**	**65**	**109**	**41**	**0.695**	**0.811**	**Include**
Sarecka-Hujar, B. (2009)	61	118	12	73	122	8	<0.001	<0.001	Exclude
**Mohamed, A. (2010)**	**20**	**37**	**43**	**2**	**11**	**37**	**0.332**	**0.516**	**Include**
**Luo, J.Y. (2014**)	**339**	**278**	**57**	**461**	**273**	**45**	**0.587**	**0.747**	**Include**
Yang, M. (2014)	305	251	48	266	160	42	0.015	0.029	Exclude

Finally included articles are shown in bold.

**Table 2:** Results of steps (i–iii) of manual HWE test

Studies	Ob = Observed genotypes				Allele frequency		Ex = Expected genotypes			X^2^	*P*-value
	KK	KE	EE	Total	K	E	KK	KE	EE		
Shang, Q. (2005)	29	33	35	97	0.47	0.53	21.3	48.3	27.3	9.75	**0.002**
Li, Y.J. (2010)	52	36	13	101	0.69	0.31	48.5	43.0	9.5	2.66	**0.103**
Lu, F.H. (2006)	45	65	59	169	0.46	0.54	35.5	83.9	49.5	8.59	**0.003**
Zhang, S.R. (2006)	69	59	13	141	0.70	0.30	68.8	59.4	12.8	0.01	**0.940**
Rao, D. (2005)	59	19	66	144	0.48	0.52	32.6	71.8	39.6	77.90	**<0.001**
Wei, Y.S. (2006)	101	103	26	230	0.66	0.34	101.1	102.8	26.1	0.00	**0.973**
Zhou, Y.L. (2006)	102	62	33	197	0.68	0.32	89.8	86.4	20.8	15.73	**<0.001**
Wang, M. (2005)	91	90	18	199	0.68	0.32	92.9	86.1	19.9	0.41	**0.524**
Jiang, H. (2002)	60	66	87	213	0.44	0.56	40.6	104.8	67.6	29.19	**<0.001**
Milutinović, A. (2006)	65	109	41	215	0.56	0.44	66.4	106.2	42.4	0.15	**0.695**
Sarecka-Hujar, B. (2009)	73	122	8	203	0.66	0.34	88.5	91.1	23.5	23.37	**<0.001**
Mohamed, A. (2010)	2	11	37	50	0.15	0.85	1.1	12.8	36.1	0.94	**0.332**
Luo, J.Y. (2014)	461	273	45	779	0.77	0.23	458.3	278.4	42.3	0.30	**0.587**
Yang, M. (2014)	266	160	42	468	0.74	0.26	255.8	180.4	31.8	5.98	**0.015**

**Table 3:** Chi-square distribution table

*P*-value	χ^2^ (df = 1)
0.995	0.000
0.975	0.000
0.20	1.642
0.10	2.706
0.05	3.841
0.025	5.024
0.02	5.412
0.01	6.635
0.005	7.879
0.002	9.550
0.001	10.828

After deleting studies with deviation from HWE and meta-analysis of included articles, we found completely different results. Genotyping data related to seven finally included articles [2–8], involving 1582 coronary heart disease (CHD) cases and 1715 controls, are shown in Table 1 (shown in bold and black color), and meta-analysis results based on five different genetics models are presented in Table 4 and Figure 1. According to our observation, we did not find a significant result in different and overall ethnicity in any genetic model. Finally, in contrast with Yin et al. [1] study and based on meta-analysis of studies in HWE, it can be concluded that ICAM-1 gene polymorphism E469K may not be related to the risk of CHD. More studies could help us to get a definitive result.

**Figure 1:**
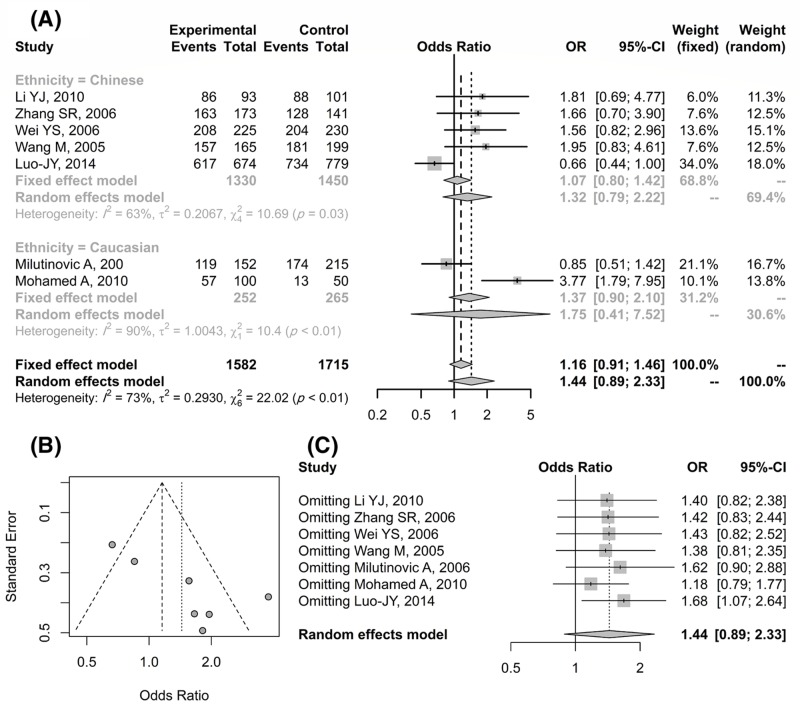
CHD risk associated with the K469E polymorphism for K/E + K/K versus E/E genotype Forest plot of CHD risk associated with the K469E polymorphism for K/E + K/K versus E/E genotype (**A**). Funnel plot (**B**) and forest plot (**C**) related to publication bias and sensitivity analysis.

**Table 4:** Meta-analysis of CHD risk associated with the K469E polymorphism based on different genetics models

Classification	Allelic (K vs. E) OR [95% CI]	*Q test P-*value	K/E + K/K vs. E/E OR [95% CI]	*Q test P-*value	KK vs. K/E + E/E OR [95% CI]	*Q test P-*value	K/E vs. K/K + E/E OR [95% CI]	*Q test P-*value
Chinese	1.23 [0.84–1.78]	0.01	1.32 [0.79–2.22]	0.03	1.25 [0.79–1.98]	0.01	0.89 [0.63–1.26]	0.01
Caucasian	1.79 [0.50–6.44]	0.01	1.75 [0.41–7.52]	0.01	2.14 [0.39–11.7]	0.03	1.26 [0.55–2.93]	0.06
Overall	1.33 [0.95–1.85]	0.01	1.44 [0.89–2.33]	0.01	1.32 [0.89–1.96]	0.01	0.95 [0.71–1.27]	0.01

Classification	K/K vs. E/E OR [95% CI]	*Q test P-*value	K/K vs. K/E OR [95% CI]	*Q test P*-value	K/E vs. E/E OR [95% CI]	*Q test P*-value		
Chinese	1.47 [0.75–2.88]	0.01	1.20 [0.78–1.83]	0.01	1.06 [0.78–1.43]	0.40		
Caucasian	2.48 [0.27–22.49]	0.01	1.19 [0.75–1.88]	0.24	1.49 [0.43–5.10]	0.01		
Overall	1.57 [0.88–2.80]	0.01	1.22 [0.86–1.74]	0.03	1.11 [0.86–1.42]	0.01		

## Abbreviations

CHD: Coronary heart disease
HWE: Hardy–Weinberg equilibrium
